# Evolutionary and Molecular Aspects of Plastid Endosymbioses

**DOI:** 10.3390/biom11111694

**Published:** 2021-11-15

**Authors:** Miroslav Oborník, Zoltán Füssy

**Affiliations:** 1 Biology Centre, Institute of Parasitology, Czech Academy of Sciences, 370 05 České Budějovice, Czech Republic; 2 Faculty of Science, University of South Bohemia, 370 05 České Budějovice, Czech Republic; 3 Faculty of Science, BIOCEV, Charles University, 128 01 Prague, Czech Republic; zoltan.fussy@natur.cuni.cz

Plastids are membrane-bound organelles that bestow phototrophic abilities to eukaryotes. They are products of endosymbiotic evolutionary events that involved eukaryotic hosts and phototrophic intracellular symbionts, represented by cyanobacteria in so-called primary plastid endosymbioses or phototrophic eukaryotes in so-called complex endosymbioses [1,2]. Plastids harbor a multitude of housekeeping and metabolic functions, directly or indirectly related to photosynthesis, which are deeply integrated with the host cell and physiology. Plastids are remarkably widespread; we know of at least 13 independent primary or complex endosymbiosis events across the tree of eukaryotes (reviewed in [3]). However, the maintenance of the photosynthetic machinery is costly; therefore, the secondary losses of photosynthesis outnumbers that of endosymbioses. Due to this deep integration with the host, plastids themselves are seldom completely lost. Their reductive evolution is gradual, with non-essential functions being lost along with photosynthesis, and other plastid features, such as thylakoids, genomic DNA, and metabolic pathways, becoming lost when they turn out to be unnecessary for the host. The complete elimination of plastids is extremely rare and has only been reported for parasitic apicomplexans (*Cryptosporidium* and gregarines) and dinoflagellates (*Hematodinium*) to date [3].

The level of complexity seen in plastids is unlikely to easily be established, and consequently, plastid proteomes manifest substantial mosaicism in terms of evolutionary origin. In line with the “shopping bag” hypothesis [4], genes encoding plastid components are recruited into the host nuclear genome from various sources, not just the immediate ancestor of the endosymbiont. These genetic “patches” are the basis of the evolutionary forces exerted to stabilize and improve the endosymbiotic partnership and make most of its potential. In this Special Issue, we collected seven articles focused on various molecular and evolutionary aspects of plastids. In one original and six review articles, we cover several plastid-bearing lineages (apicomplexan-like parasites, chlorarachniophytes, archaeplastids, dinoflagellates, and diatoms), facets of plastid biology (DNA replication, codon adaptation, RNA editing, nucleotide transport, light-harvesting complexes), and evolutionary insights into innovation, non-adaptive traits, and functional reduction. 

A general review concerning the constructive evolution of plastid endosymbiosis, followed by the reductive evolution of plastids leading to secondary heterotrophy and parasitism, introduces this Special Issue [3]. This is followed by a comprehensive overview of reductive plastid evolution, as exemplified by Apicomplexa and their relatives sensu lato [5]. Eric Salomaki and Martin Kolísko introduce the apicoplast, the relic plastid of apicomplexan parasites (e.g., *Plasmodium*, *Toxoplasma*), and summarize current knowledge about its origin, with special emphasis on the early branching gregarines and corallicolids, a recently described group of non-photosynthetic coral symbionts that still encode part of the chlorophyll synthesis pathway in their plastid genomes [5,6,7]. In addition, they focus on the sister groups to apicomplexan parasites, including chromerids [8], their close parasitic relative *Piridium sociabile* [9], and the early-branching, gregarine-like parasite *Platyproteum* [9]. An original research article from this Special Issue focuses on organellar DNA polymerases in algae with complex plastids to illustrate that the evolution of plastids is tightly linked to the evolution of mitochondria via gene acquisitions and protein retargeting [8]. Yoshihisa Hirakawa and Arisa Watanabe [10] show that chlorarachniophytes encode two evolutionarily distinct nucleus-encoded plant and protist organellar DNA polymerases (POPs) targeted to the secondary plastids and mitochondria, respectively. Dinoflagellates also possess two POP genes, in contrast with haptophytes and cryptophytes, which have only one POP. The functional recruitment of this replicase is, therefore, lineage-specific [8]. Two reviews in the Special Issue spotlight diatoms [11,12]. Tomomi Nonoyama and colleagues [11] present a comprehensive transcriptomic analysis of metabolism in diatoms, a group of complex plastid-bearing algae that dominate the world’s oceans. They identified several metabolic innovations specific to diatom plastids, for example, the ornithine cycle, iron acquisition proteins, or tocopherol synthesis. They also investigate the metabolism of the diatom *Fistulifera solaris* investigated for biotechnology-scale biofuel production. Ansgar Gruber and Ilka Haferkamp [12] reviewed the metabolism and transport of nucleotides in diatoms. Their review mainly focuses on the function, diversity, and origin of nucleotide transporters (NTTs). Despite the host/eukaryotic origin of NTTs in diatoms, nucleotide transporters facilitated the endosymbiotic evolution of plastids and biotechnological development of synthetic organisms [12]. Christen Klinger and Elisabeth Richardson explore the development of plastid genomics in the high-throughput sequencing era. They propose that publicly available plastid genomic data can be used, for example, to further test both previously formulated and new hypotheses about the evolution of plastid features, such as codon adaptation, transcript editing, and the “limited transfer window” hypothesis, across the diversity of uncultured eukaryotes. The advancement of novel computational methods and machine learning, and accumulation of new sequence data promises exciting discoveries in the future [13]. Last but not least, Beverley Green analyzes and describes the intriguing fate of phycobilisomes (PBS), the light-harvesting complexes of cyanobacteria, rhodophytes, and glaucophytes, which were completely replaced in the green lineage and the rhodophyte-derived complex plastids [14].

This Special Issue of *Biomolecules* brings fresh insights into the evolutionary and molecular aspects of endosymbiosis, leading to the appearance and loss of plastids. We wish our readers an inspiring time reading.
